# Ex vivo radiosensitivity is increased in non-cancer patients taking valproate

**DOI:** 10.1186/s12883-020-01966-z

**Published:** 2020-10-24

**Authors:** Jenny Stritzelberger, Jennifer Lainer, Stefanie Gollwitzer, Wolfgang Graf, Tina Jost, Johannes D. Lang, Tamara M. Mueller, Stefan Schwab, Rainer Fietkau, Hajo M. Hamer, Luitpold Distel

**Affiliations:** 1 Department of Neurology, Universitätsklinikum Erlangen, Friedrich-Alexander-Universität Erlangen-Nürnberg (FAU), Erlangen, Germany, Schwabachanlage 6, 91054 Erlangen, Germany; 2 Department of Radiation Oncology, Universitätsklinikum Erlangen, Friedrich-Alexander-Universität Erlangen-Nürnberg (FAU), Erlangen, Germany, Universitaetsstraße 27, 91054 Erlangen, Germany

**Keywords:** Valproate, Individual radiosensitivity, 3-colour fluorescence-in-situ-hybridization, Breaks per metaphase

## Abstract

**Background:**

Valproate (VPA) is a commonly prescribed antiepileptic drug for patients experiencing epileptic seizures due to brain tumors. VPA increases radiation sensitivity in various tumor cells in vitro due to complex mechanisms. This could make tumors more vulnerable to ionizing radiation or overcome radioresistance. Yet, clinical data on possible improvement of tumor control by adding VPA to tumor therapy is controversial. Potentially radiosensitizing effects of VPA on healthy tissue remain unclear. To determine individual radiosensitivity, we analyzed blood samples of individuals taking VPA.

**Methods:**

Ex vivo irradiated blood samples of 31 adult individuals with epilepsy were studied using 3-color fluorescence in situ hybridization. Aberrations in chromosomes 1, 2 and 4 were analyzed. Radiosensitivity was determined by the mean breaks per metaphase (B/M) and compared to age-matched (2:1) healthy donors.

**Results:**

The patient cohort (*n* = 31; female: 38.7%) showed an increase of their average B/M value compared to healthy individuals (*n* = 61; female: 56.9%; B/M: 0.480 ± 0.09 vs. 0.415 ± 0.07; *p* = .001). The portion of radiosensitive (B/M >  0.500) and distinctly radiosensitive individuals (B/M >  0.600) was increased in the VPA group (54.9% vs. 11.3 and 9.7% vs. 0.0%; *p* < .001). In 3/31 patients, radiosensitivity was determined prior to and after VPA treatment and radiosensitivity was increased by VPA-treatment.

**Conclusions:**

In our study, we confirmed that patients treated with VPA had an increased radiosensitivity compared to the control group. This could be considered in patients taking VPA prior to the beginning of radiotherapy to avoid toxic side effects of VPA-treatment.

## Background

Valproate (VPA) is a commonly prescribed antiepileptic drug. Besides its antiseizure property, VPA is an effective inhibitor of histone deacetylase (HDAC) which is involved in modulating chromatin structure and gene expression [1, 2]. Like other HDAC inhibitors [3–5], VPA has also been shown to enhance the radiosensitivity of a variety of tumor cell types [6–8], including glioma cell lines [9–12]. However, the exact mechanisms are complex and not fully understood [13]. While the radiosensitizing effects seem clearly evident in vitro and potential benefits for oncological treatment by rendering tumor cells more vulnerable to irradiation and overcoming radioresistance seem plausible [6, 14], evidence in clinical studies is sparse and controversial and tumor control was not improved by adding VPA to therapy regimes [15, 16].

However, potentially radiosensitizing effects of VPA on healthy tissue remain unclear. It is well known that enhanced radiosensitivity is associated with an increase of both acute and late adverse effects of irradiation due to various causes such as certain drugs like chloroquine or vemurafenib and diseases like Ataxia teleangiectasia or Nijmegen breakage syndrome in patients undergoing radiation treatment [17–20]. In these cases, a dose reduction is recommended for distinctly radiosensitive patients [21, 22].

Radiotherapy-associated adverse effects not only impact patient’s quality of life but also potentially lead to discontinuation of therapy and even life-threatening conditions [23, 24]. An increase in adverse effects due to high radiosensitivity would be a possible explanation for the phenomenon that VPA shows beneficial effects on tumor therapy in vitro but many clinical studies do not significantly show this effect. We hypothesize that VPA treatment leads to enhanced radiosensitivity in patients’ healthy blood cells while also increasing radiosensitivity of tumor cells in vitro.

To determine individual radiosensitivity, we analyzed chromosomal aberrations in lymphocytes of blood samples of individuals with epilepsy without tumors but taking VPA using the three-color-FiSH.

## Methods

### Patients

Whole blood samples (NH4-Heparin, Sarstedt, Nürnbrecht, Germany) of 31 patients with VPA were drawn. Sixty two healthy age-matched individuals from a collective previously published served as controls [25, 26]. For this study, individuals having any other medical condition or taking drugs suspected to increase radiosensitivity like chloroquine, efavirenz, nelfinavir, vemurafenib, metformin, or immunotherapy [18, 27–30] were excluded as well as individuals suffering from any malignant neoplasms. Patients were consecutively sampled at the epilepsy center of the university hospital of Erlangen-Nürnberg. This study was approved by the ethics review committee of the Friedrich-Alexander-Universitaet Erlangen-Nuernberg (No. 21_19B). Written informed consent was obtained from all the patients.

#### Chromosome preparation and three color fluorescence in situ hybridization

VPA trough levels were analyzed as well as the VPA level at the time of the blood collection. The three color fluorescence in situ hybridization (FiSH) assay was performed to study radiation sensitivity. The blood of each individual was divided into two portions One portion served as the control sample to detect spontaneous aberrations and was sham irradiated, while the other portion was irradiated with a dose of 2 Gy by a 6-MV linear accelerator (Mevatron, Siemens, Germany). Two Gy corresponds to the dose per day patients receive during radiotherapy. The lymphocytes were stimulated with phytohemagglutinin (Biochrom, AG, Berlin, Germany) and cultured in an incubator at 37 °C. Lymphocytes were grown for 48 h and blocked in the metaphase of the first cell division by colcemid (Gibco, Waltham, MS, USA). Subsequently, chromosome preparations were performed. To detect chromosome 1, 2 and 4, the DNA was hybridized with chromosome specific probes. Staining was performed using different fluorescent dyes. Chromosomes were counterstained with DAPI. The three color fluorescence in situ hybridization was implemented as previously described [31, 32].

#### Image acquisition and analysis

To search chromosome metaphase spreads automatically at 100× magnification, a fluorescence microscope (Zeiss, Axioplan 2, Göttingen, Germany) and the Metasystems software (Metafer 4 V3.10.1, Altlussheim, Germany) were used. An image of each metaphase was acquired at a magnification of 630×. For each metaphase spread black and white images of each color (red, green and blue) were acquired and used for evaluation. Using an image analysis software (Biomas, Erlangen, Germany), at least 200 metaphases were analyzed. Translocations, dicentric chromosomes, acentric chromosomes, rings, deletions, insertions and complex chromosomal rearrangements (CCRs) were scored. Subsequently, the data were transferred to a spreadsheet (Excel, Microsoft Corporation, Redmond, WA, USA) and scores (breaks per metaphase, B/M) were calculated. The aberrations were scored by the number of underlying chromosomal breakages according to Savage and Simpson [33]. The B/M value of the irradiated sample was corrected by the B/M value of the sham irradiated sample.

#### Drugs

VPA was obtained from Enzo (Farmingdale, NY, USA) and was dissolved in dimethyl sulfoxide (DMSO; Carl Roth, Karlsruhe, Germany). Stock solutions were stored at − 20 °C.

### Statistical analysis

The statistical analysis of the data was performed using SPSS Statistics 22.0 (IBM, Armonk, NY, USA) [29, 30]. Levene-test and the two-sided T-test were used to test for significant differences between both groups, Pearson’s r correlation was calculated to test possible correlations and exact Fisher’s test to compare the distribution of different groups of radiosensitivity. Shapiro-Wilk-test was used to test for normality. *P*-values < 0.05 were regarded as significant.

## Results

### VPA patients and healthy controls

In total, blood of 93 individuals of different ages was studied for radiosensitivity by FiSH. The whole cohort consisted of 31 patients and 62, age-matched healthy donors (Table 1). The mean age was 46.4 years (range 21–84 a). 38.7% of the patients were female. The average dosage of VPA patients received per day was 1700 mg (range: 900–3000 mg), plasma trough levels were 65.5 mg/l compared to blood levels of 64.8 mg/l directly at the time of blood withdrawal. Patients took between 0 and 10 other drugs in addition to VPA (average 3.7, Table 1). The indication for VPA was mostly epilepsy (90.0%), whereas 3 patients received VPA due to bipolar disorder. In the epilepsy group, 14 patients had partial onset seizures, 8 patients had seizures with generalized beginning. In 4 patients, VPA was used to treat status epilepticus. Three of the 31 patients were either started on VPA or the prescription was stopped so it was possible to obtain samples with or without VPA in the same patient.

**Table 1:** Characteristics of healthy individuals and VPA-Patients

	Healthy Individuals					Patients				
Variable	*n*	mean	SD	min	max	*n*	mean	SD	min	max
Age (years)	62	46.4	17.4	21	84	31	46.4	17.5	21	84
Sex (% female)	62	56.9	–			31	38.7	–	–	–
Dosage per day (mg)	–	–	–	–	–	31	1708	637.1	900	3000
Height (cm)	–	–	–	–	–	18	175.7	10.1	158	190
Weight (kg)	–	–	–	–	–	27	83.4	17.9	50	126
Trough plasma level (mg/l)	–	–	–	–	–	27	65.5	20.0	24.6	99.8
Plasma level at time of blood draw (mg/l)	–	–	–	–	–	29	64.8	21.6	20.0	108.0
Epilepsy (%)	–	–	–	–	–	31	90.0	–	–	–
Comedication	–	–	–	–	–	31	3.7	3.0	0	0

### B/M values in patients taking VPA and healthy donors

**Fig. 1:**
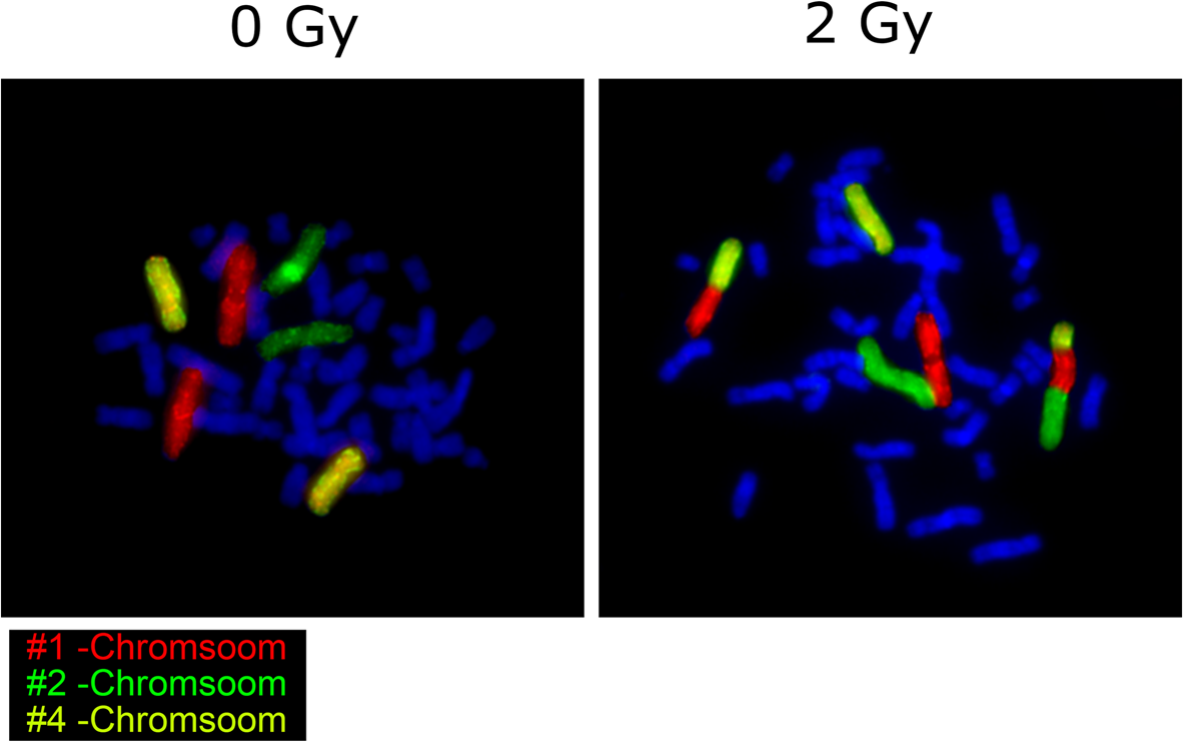
Three-color-FiSH. Three-color fluorescence in situ hybridization to analyze radiosensitivity. Metaphase spreads of human blood lymphocytes, stained with chromosome specific probes for chromosome # 1 (red, rhodamine), chromosome # 2 (green, FITC) and chromosome # 4 (yellow, rhodamine + FITC). DNA was stained with DAPI (blue)

**Fig. 2:**
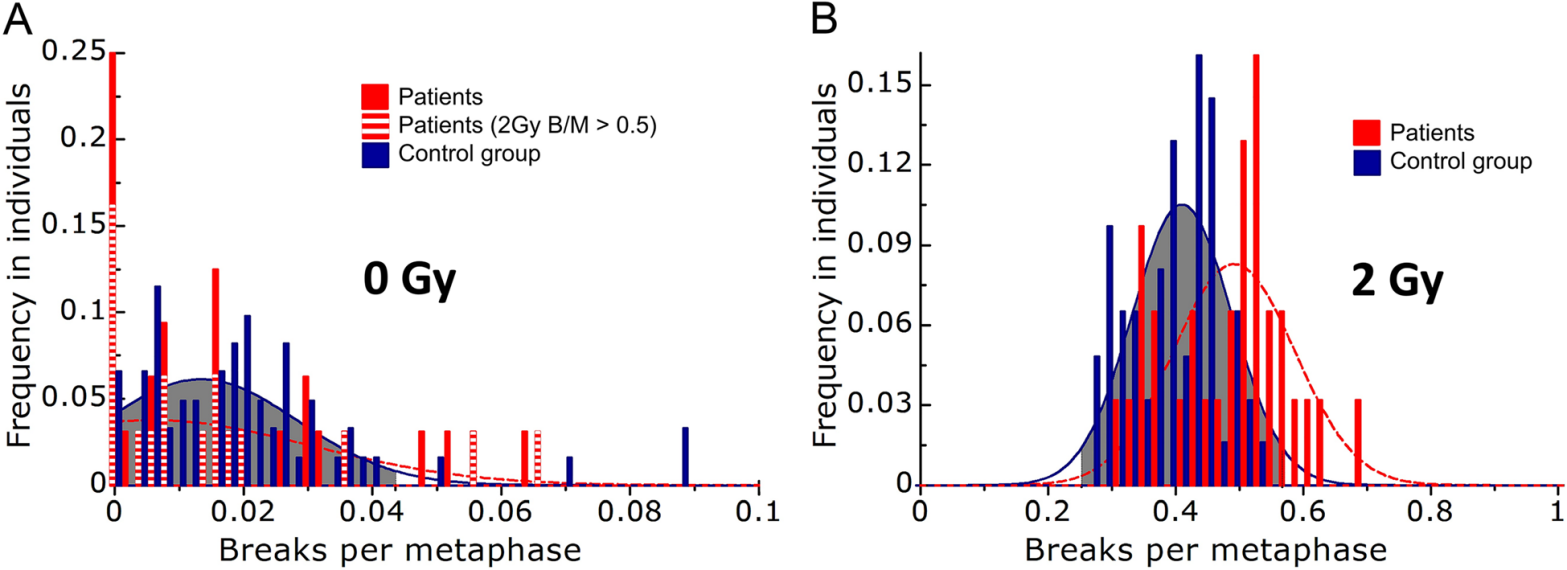
Radiosensitivity in patients taking VPA vs. healthy individuals. Number of healthy individuals (blue) and patients (red) classified into divisions of 0.02 breaks per metaphase. B/M background without irradiation **a** and after irradiation with 2 Gy, corrected by the background **b**. Radiosensitive patients with a B/M-value at 2 Gy > 0.5 are additionally labelled to show that these patients did not have more spontaneous aberrations (A). The data were fitted by a Gaussian distribution; scale 10 μm

Radiosensitivity was studied by a three-color-FiSH (Fig. 1). The average aberration frequency is expressed as breaks per metaphase (B/M). All B/M-values at 2 Gy were corrected by their background levels (i.e. the B/M-values of the sham irradiated samples). We did not detect any differences for the B/M-values at 0 Gy, which shows that the rate of spontaneous chromosomal aberrations did not differ between patients and healthy individuals and especially not between radiosensitive patients and other individuals tested (B/M 2 Gy >  0.5) (Fig. 2 a). The patient cohort’s average B/M value was clearly increased compared to healthy individuals (patients: 0.480 ± 0.09 vs.healthy individuals: 0.415 ± 0.07; *p* = .001) (Table 2). B/M-values were approximately normally distributed for healthy individuals (*p* = .077) and for patients (*p* = .311), as assessed by the Shapiro-Wilk-Test, Fig. 2b.

**Table 2:** B/M-values after ex vivo irradiation by 2 Gy in patients and healthy individuals

	Healthy Individuals (n)	Patients (***n***)	*p*-value
Mean B/M-value (2 Gy)	0.415 ± 0.07 (62)	0.480 ± 0.09 (31)	.001
< 0.500 B/M	88.7% (55)	45.2% (14)	<.001
> 0.500 B/M	11.3% (7)	54.9% (14)	<.001
> 0.600 B/M	0.0% (0)	9.7% (3)	<.001

Mean values of breaks per metaphase are given with standard deviations. Fractions of healthy individuals vs. patients with average B/M (B/M ≤ 0.500), increased (B/M > 0.500) and distinctly increased radiosensitivity (B/M > 0.600) are given

Additionally, we studied the distribution of B/M values in the healthy individuals and cancer patient cohort. In previous studies we found that from a value of 0.5 B/M on an enhanced radiosensitivity can be assumed and from a value of 0.6 B/M on an individual is considered to have a distinctly increased radiosensitivity [31]. The portion of radiosensitive (B/M >  0.5) and distinctly radiosensitive individuals (B/M > 0.6) were both noticeably increased in the VPA group (*p* < .001, Table 2).

### B/M values in three patients before and during treatment with VPA

In 3 of the 31 patients, we were able to compare radiosensitivity in the same patient under VPA treatment versus dose reduced or VPA-free intervals (Table 3). One patient was started on VPA and his radiosensitivity was determined prior to initiation (VPA-dosage per day: 0 mg) and 72 h after beginning of treatment (VPA-dosage per day: 2400 mg). The second patient’s dose was significantly reduced during video-EEG monitoring in order to provoke seizures (VPA-dosage: 0 mg vs. 3000 mg). The third patient was put on drug holiday to provoke seizures during video-EEG monitoring for 3 consecutive days also in a monitored setting (VPA-dosage: 500 mg vs. 1500 mg). Taken together, although the settings were different for each patient, it was apparent that radiosensitivity was increased by VPA-treatment in all of these three patients.

**Table 3:** Profiles of three patients and their radiosensitivity under different settings of VPA intake

	Patient 1	Patient 2	Patient 3
VPA-dosage per day (1) (mg)	0	0	500
VPA-dosage per day (2) (mg)	2400	3000	1500
B/M-value 2 Gy (1)	0.491	0.399	0.354
B/M-value 2 Gy (2)	0.576	0.522	0.630
Distance between time point (1) and (2)	72 h	48 h	72 h
Age	78	35	21
Sex	M	M	M
Height (cm)	n.a.	173	n.a.
Weight (kg)	80	72	86
Comedication	10	2	2

(1) Is the first time point with the specific VPA dosage and the first determination of radiation sensitivity and (2) is the second time point with the VPA dosage and the second determination of radiation sensitivity in identical patients

### Co-dependencies

Furthermore, patient’s age, weight, dosage of VPA, dosage per kilogram bodyweight, trough and plasma levels, and number of comedications and the 2 Gy B/M values of the patient cohort were analyzed for possible correlations. We could not detect significant correlations between any of these characteristics (Pearson: age r = .82, *p* = .435, weight r = −.192, *p* = .330, dosage r = .173, *p* = .353, dosage per kg r = .234, *p* = .240, trough level − .153, *p* = .503, level at blood draw −.358, *p* = 0.56, number of comedication .048, *p* = .798). Additionally, we checked the plasma concentration in the VPA-groups with a B/M-value < 0.500, > 0.500 and > 0.600 and did not find a significant correlation between degree of change in radiosensitvitiy and plasma concentration (r = .016, *p* = .937).

## Discussion

In this study, we found that VPA has radiosensitizing effects on peripheral blood lymphocytes ex vivo.

In order to analyze chromosomal aberrations after ex vivo irradiation, we used the 3C-FiSH. Studying chromosomal aberrations has been used for many years to anticipate patient’s radiosensitivity with the aim to individualize dose or fractionation in clinical radiotherapy [34–36]. Lymphocytes of the peripheral blood are especially suitable as they are in the G0-phase of the cell cycle thus avoiding cell-cycle-dependent differences in radiosensitivity. After irradiation, cells need to pass through various damage processing and control functions. Having passed these checkpoints, lymphocytes enter metaphase; if one of these is impaired, however, increased radiosensitivity may occur. As chromosomal aberration analysis tests for the most important cellular functions, this late endpoint has shown potential to predict radiosensitivity [37, 38]. In addition, it is assumed that radiation sensitivity is genetically determined and that it is therefore possible to infer from the radiation sensitivity of lymphocytes to all body cells and also to tumor cells [26, 39]. This makes it possible to adjust the radiation therapy dose to the radiation sensitivity of the patient [40, 41]. However, this procedure still has experimental value.

We studied a healthy individual cohort and a patient cohort taking VPA on a daily regular base. Since age is the only factor in the patient’s characteristic of which we are aware that it may affect radiosensitivity [26], we did only match the cohorts for this item. In the VPA patient cohort a considerable portion of individuals with increased radiosensitivity was expected, due to already well-known radiosensitizing properties of VPA as a HDAC-inhibitor [9–13]. In our study, we found not only that patients taking VPA were clearly more radiosensitive than healthy controls but also the rate of increased radiosensitive (B/M value > 0.5) and distinctly radiosensitive (B/M > 0.6) individuals were increased in the VPA group. As radiosensitivity may differ inter-individually, we also studied radiosensitivity in three patients at two different time-points with various doses of VPA which showed that VPA-induced radiosensitivity depends on the presence of VPA in the cell and after it is no longer existent it has no more effect. This is interesting because the differences were measured with only a few days in between which supports the view that VPA-induced radiosensitivity is a short-term-effect and quickly decreases after treatment is stopped.

We studied various potential confounders that could possibly influence the intensity of the radiosensitizing effect of VPA, such as age [26], dosage of VPA, trough levels and plasma levels at blood withdraw, dosage per kilogram, and number of comedications. Because time between taking VPA and blood drawing varied, we also examined VPA levels at the time of the blood sampling. However, none of these characteristics significantly correlated with the B/M-values. This might be explained by the fact that the pre-existing, underlying individual radiosensitivity is variable [21] and could have had more impact as a confounder than external factors; another possible explanation would be that there is a certain threshold from which on VPA induces dose-independent radiosensitivity. However, the number of patients was limited in this study and further research with larger case numbers would be desirable to investigate potential influencing.

There is abundant in vitro evidence on radiosensitizing effects of VPA on many different tumor cell lines; this leads to the assumption that adding VPA would lead to improved tumor control by making cancer cells particularly susceptible to irradiation, overcoming potential radioresistance and thus better outcomes. Despite promising preclinical results, however, evidence for clinical benefit of VPA in oncological treatment remains controversially discussed [15, 42–44]. We hypothesize that patients taking VPA might have an increased risk to suffer from radiation induced side effects due to their increased radiosensitivity. These effects may possibly balance out beneficial effects of VPA on tumor growth and therapy thus resulting in non-significant findings. Clinical data on adverse effects under concurrent therapy with VPA and irradiation would be desirable to support this hypothesis.

There are certain limitations of this study. For instance, we did not check the free fraction of VPA which might have been different from the values we detected due to changes in plasma albumin binding. There is missing data on body height and weight in a few patients because patients did not know or did not want to indicate this information and in a few patients, plasma levels could not be examined due to logistic reasons. Although comedication did not correlate with radiosensitivity in our study, we cannot exclude that it may have influenced radiosensitivity since the healthy individuals did not take any comedication. To our knowledge, however, there is no data on the subject if taking medication in general leads to an increase in radiosensitivity and we do not know of any mechanisms by which medication in general would affect radiosensitivity on the DNA-level. However, further research is needed to elucidate this issue.

## Conclusions

In our study, we confirmed that non-cancer patients treated with VPA had an increased radiosensitivity compared to the control group. This could be considered in patients taking VPA prior to the beginning of radiotherapy to avoid toxic side effects of VPA-treatment.

## Data Availability

The datasets used and/or analyzed during the current study are available from the corresponding author on reasonable request.

## Abbreviations

B/M: Breaks per metaphase
DAPI: 4′,6-diamidin-2-phenylindol
FiSH: Fluorescence in situ hybridization
HDAC: Histone deacetylase
VPA: Valproate
